# Current status survey of the extramural hospital management of venous thromboembolism after total hip and knee arthroplasty in China

**DOI:** 10.1186/s12891-021-04663-1

**Published:** 2021-09-13

**Authors:** Kai Tong, Hankun Liu, Jun Qin, Zhengqi Pan, Yangfan Shangguan, Hao Xiao, Hua Wang, Liaobin Chen, Yang Tan

**Affiliations:** Division of Joint Surgery and Sports Medicine, Department of Orthopedic Surgery, Zhongnan Hospital of Wuhan University, 169 Donghu Road, Wuhan City, Hubei Province 430071 PR China

**Keywords:** Venous thromboembolism, Major orthopaedic surgery, Quality improvement, Satisfaction

## Abstract

**Background:**

Venous thromboembolism (VTE) is a potentially fatal complication after arthroplasty. Numerous prophylactic strategies and studies to reduce VTEs have focused on the duration of the hospital stay and on few extramural hospitals. This study aimed to investigate extramural hospital management of VTE after total hip/knee arthroplasty (THA/TKA) in China with a novel survey tool.

**Methods:**

A total of 180 patients undergoing arthroplasty, including 68 THA patients and 112 TKA patients, were enrolled in this study. All patients received anticoagulant treatment management. A survey querying VTE management and adherence, such as therapy information, understanding of anticoagulation, satisfaction with the ability of medical staff, and satisfaction with health care costs, was administered by a questionnaire (TKA/THA Patients’ Experience with Anticoagulation in the Post-discharge Period) for quality improvement.

**Results:**

The average age of the patients was 65.27 ± 13.62 years. All patients knew their follow-up times. 85 % of them were suggested that re-examine at the next 14 days, and the others at the next 28 days. All patients continued to visit the orthopaedic clinic after discharge without choosing other types of outpatient services, such as an anticoagulant clinic or home visit with a nurse/pharmacist or remote evaluation by telephone. A total of 96.6 % of all patients used new oral anticoagulants, and the most common treatment duration was 2–4 weeks (93.3 %). 48 % informed their physicians that they were taking anticoagulation medications when they visited ophthalmology, dentistry, dermatology, and other departments. The overall rate of satisfaction with anticoagulation management was 81.67 %, and 6.67 % of patients were not unsatisfied with their medical expenses. Patient compliance decreased with increasing follow-up time. Continuous follow-ups after discharge significantly improved patient compliance.

**Conclusions:**

These results elucidate how we can improve the quality of anticoagulation. Continuous follow-up appointments for 30 days after discharge, especially for individuals over 65 years old, significantly improved patient satisfaction and reduced the incidence of VTE and medical costs.

**Supplementary Information:**

The online version contains supplementary material available at 10.1186/s12891-021-04663-1.

## Key Messages

1. This study examines the current status of extramural hospital prevention and treatment of VTE after total hip/knee arthroplasty. This study aims to provide high-quality evidence for future clinical investigation via a randomized controlled trial.
2. A novel tool (TKA/THA Patients’ Experience with Anticoagulation in the Post-discharge Period) for quality improvement in anticoagulation management after THA/TKA discharge was designed to assess the treatment process and patient treatment satisfaction.
3. Continuous follow-up appointments for 30 days after discharge, especially for individuals over 65 years old, significantly improved patient satisfaction and reduced the incidence of VTE and medical costs.

## Background

Despite considerable advances in surgical and anaesthetic techniques, venous thromboembolism (VTE) is still a common complication following major orthopaedic surgery [1, 2]. The incidence of VTE ranges from 35 to 84 % of patients undergoing total hip and knee arthroplasty (THA/TKA) [1, 3–5]. VTE has become a severe postoperative complication leading to death in the perioperative period and unexpected deaths. In these patients undergoing TKA, the incidence rate of VTE after the operation, including symptomatic deep vein thrombosis (DVT) and pulmonary embolism (PE) [4], is approximately 0.45-5.30 %, while the rate of VTE in patients undergoing THA is approximately 0.24-1.60 % [6–9]. Although the overall DVT rates after TKA/THA have reduced over the past decade, the number of patients in the USA has increased with the rising amount of surgery [6]. However, up to 2016, Chinese patients still presented a higher frequency of VTE (after THA, 2.4-6.5 %; after TKA, approximately 3.19 %) [2]. Consequently, the quality of VTE management after TKA/THA should be improved. Reasonable and practical preventive measures and long-term management are essential means to prevent VTE [10].

Many studies on clinical guidelines for VTE prophylaxis are intended to assess the management of patients undergoing THA/TKA in the hospital [1, 3, 11]. However, there are quite a few VTEs that occur in outpatients after surgery. Among those patients with VTE, approximately 57.9 % of THA patients and 38.3 % of TKA patients are observed in the post-discharge period [12]. In addition, many uncontrollable factors lead to the failure of treatment and the increase in VTE incidence during anticoagulation out of the hospital. Patients at home may often face this situation of no supervision by doctors or nurses in time. Thus, the misunderstanding of VTE disease, unawareness of therapy importance, and low compliance may result in higher incidences of VTE [11].

For these reasons, a novel tool (TKA/THA Patients’ Experience with Anticoagulation in the Post-discharge Period) for quality improvement of VTE management was designed. Validated by five medical centres in China, the device has been used for six years to follow patients’ living status. We collected information on the current status of VTE prevention after hip and knee arthroplasty in China, such as anticoagulant treatment, understanding of the disease, satisfaction with the ability of medical staff, and satisfaction with health care costs. Thus, this study provides essential data to further improve the quality of medical services for anticoagulation after TKA/THA outside the hospital and decrease the risk of VTE disease.

## Methods

### Study material

To obtain the required data to test the proposed tool, patients who underwent TKA/THA by the same team of surgeons from April 2017 to June 2019 and were subsequently prescribed an anticoagulant to prevent VTE were identified in our medical centre. For patients with confirmed or highly suspected lower-extremity proximal DVT provoked by total hip/knee arthroplasty, we routinely designed a treatment protocol selected for anticoagulation according to the 9th VTE treatment guidelines [13]. Using the novel tool for quality improvement (TKA/THA Patients’ Experience with Anticoagulation In the Post-discharge Period) to collect patient data on basic information, treatment process data, understanding of anticoagulation, and patient assessment of treatment satisfaction. Our hospital’s ethics committee approved the present study in December 2018, and all patients signed informed consent forms.

### Inclusion criteria

(1) Patients undergoing total primary knee or hip arthroplasty, (2) patients prescribed an anticoagulant to prevent VTE, and (3) patients older than 18 years of age.

### Exclusion criteria

(1) Patients receiving anticoagulation for an indication other than VTE prevention, (2) patients who had dementia, cognitive impairment or pregnancy, (3) patients for whom anticoagulation was contraindicated, and (4) additional inclusion/exclusion criteria from the customer.

### DATA & the novel tool for quality improvement

The novel tool consisted of (1) basic information of the patients, (2) follow-up and therapy, (3) patient knowledge about VTE disease, (4) treatment outcome and satisfaction, (5) warfarin usage information, and (6) self-administered injection treatment. The patients completed the form on the first return visit. Data were in the form of self-reported non-identifiable data. The data were aggregated and analysed using Microsoft Access or Excel. The questionnaire was formatted as a scanned-form data collection tool and was used as the survey instrument to gather the data from the participants. (See Appendix A).

### Statistical analysis

We presented descriptive statistics and bivariate analyses to calculate the frequency and percentages for all variables in the questionnaire. A chi-square test was applied to compare differences in rates among different groups as follows: “compliance data”. All data analyses were conducted with SPSS 22.0 software (SPSS Inc., Chicago, IL). Statistically, we defined a significant difference as a P value of less than 0.05.

## Results

### Basic information of VTE patients and prophylaxis

A total of 180 patients were enrolled in this study: 35 % (63) patients were male, and 65 % (117) were female. The mean age of the patients was 65.27 ± 13.62 years; males were 65.25 ± 13.98 years old, and females were 65.3 ± 13.62 years old. Our main survey contents involved the follow-up appointment time, type of hospital revisited, the type and timing of the anticoagulation protocol, and the supervised situation during anticoagulant treatment. The detailed data are shown in Table 1.

**Table 1:** the information reexamines and therapy

Demographic	number(percent %)
Appointment scheduled within (day)	
7 d	0
14 d	153(85 %)
21 d	0
28 d	27(15 %)
I didn’t have an appointment scheduled	0
managed by a(n)	
Anticoagulation Clinic	0
Doctor’s Office	180(100 %)
At home with visiting nurse/pharmacist	0
Remotely by telephone	0
At another type of site	0
After discharge, I received these anti-clotting drugs (check all that apply)	
Vitamin K Antagonist	0
New Oral Anticoagulant	174(96.6 %)
Heparin Sodium Injection	3(1.7 %)
Aspirin	3(1.7 %)
Others	0
I have been on my anti-clotting drug for	
less than 3 days	0
3days–1 week	0
2–4 weeks	168(93.3 %)
4–5 weeks	9(5.0 %)
more than 5 weeks	3(1.7 %)
When see the eye doctor, dentist, skin doctor, etc., tell them that I am on an anti-clotting drug	
All or some	87(48 %)
no	93(52 %)
Admitted to an ER or hospital with taking your anti-clotting drug?	
Yes	0
No	180(100 %)

All follow-up durations of the VTE patients were recorded. 85 % of patients were re-examined in the next 14 days, and the others were re-examined in the next 28 days. Every patient continued to visit the orthopaedic clinic after discharge without choosing an anticoagulant clinic or community medical institution or receiving a diagnosis and treatment plan by telephone. A total of 174 (96.6 %) of all patients used new oral anticoagulants, 3 (1.7 %) used injection anticoagulants, and 3 (1.7 %) took aspirin. Most patients (93.3 %) took anticoagulants after surgery for 2–4 weeks; 5.0 %, for 4–5 weeks; and 1.7 %, for more than 5 weeks. 48 % informed their doctors that they were taking anticoagulation medications when visiting ophthalmology, dentistry, dermatology, and other departments. Among them, 48 patients were younger than 65 years old, and 39 were older than 65 years old. There was no patient hospitalization because of anticoagulation management. The rate of overall satisfaction with anticoagulant treatment was 81.67 %, and 6.67 % of patients were not unsatisfied with their medical expenses. Notably, during the one-month follow-up period, no obvious VTE events or related complications were found in any cases.

### Understanding VTE disease and anticoagulant treatment ability

All the patients attended a meeting for patient education before discharge. At that time, they were asked and surveyed regarding their understanding of VTE disease and the ability of anticoagulant treatment during their follow-up visit. The results showed that more than 75 % of the patients had a clear understanding of the diagnosis of VTE disease, which activities to avoid and when to call the clinic or doctor. Approximately 50 % knew the benefits of therapy, as well as how much of the drug to take and when to take it. A total of 30-49 % knew who to call if there was a problem as well as the importance of taking their medication. However, less than 30 % carefully read the anticoagulant instructions, including indications, contraindications, and side effects of the drugs. Even less than 10 % obtained the follow-up treatment plan and knew how long anticoagulant therapy would be sustained. Indeed, patients inevitably were confronted with more complicated situations including but not limited to the following: signs and symptoms of easily bleeding, what to do if missing a dose, what to do if he or she happened to undergo a dental treatment procedure, whether haemostatic drugs were available to treat bleeding or not, and other drugs or foods that could interact with his or her anticoagulant drug.

Patients younger than 65 years old (n = 81) had more knowledge and awareness of VTE disease than those older than 65 years old. Of all 17 items in the survey tool, the average number of known items was 8 for patients younger than 65 and 4.8 for more than 65 (n = 99) (Table 2).

**Table 2:** The results of understanding of the disease and the ability of anti-clotting

Demographic	(*n* = 180) n(%)		≤ 65(*n* = 81) n(%)	> 65(*n* = 99) n(%)
Knowing diagnosis		153(85 %)	72(88.9 %)	81(81.8 %)
Knowing benefits of therapy		87(48 %)	51(63 %)	36 (36.7 %)
Knowing who to call if there is a problem		72(40 %)	42 (51.9 %)	30 (30.3 %)
Knowing activities to avoid		129(72 %)	66 (81.5 %)	63 (63.6 %)
knowing when to call the clinic or doctor		129(72 %)	66 (81.5 %)	63 (63.6 %)
Knowing signs and symptoms of bleeding		39(22 %)	30 (37 %)	9 (9 %)
Knowing side effects		36(20 %)	21 (26 %)	15 (15.2 %)
Knowing what to do if miss a dose		21(12 %)	15 (18.5 %)	6 (6 %)
Knowing how much drug to take and when		117(65 %)	78 (96.3 %)	39 (39.4 %)
Knowing importance of taking my medicine		72(40 %)	45 (55.6 %)	27 (27.3 %)
Knowing how long I will need to take and when		96(53 %)	66 (81.5 %)	30 (30.3 %)
Knowing what to do if I am having a dental or medical procedure		12(7 %)	6 (7.4 %)	6 (6 %)
Knowing my medication list including any changes		72(40 %)	54 (66.7 %)	18 (18.2 %)
Knowing risks if I take too little or too much		15(8 %)	6 (7.4 %)	9 (9.1 %)
Knowing other anti-clotting drugs available for use in treating a clot		30(17 %)	18 (22.2 %)	12 (12.1 %)
Knowing the affect food can have on the effectiveness		18(10 %)	15 (18.5 %)	3 (3 %)
Knowing other drugs and over-the counter products that can interact with my anti-clotting drug		21(17 %)	15 (18.5 %)	6 (6 %)

### Patient compliance

As shown in Table 3, female patients had significantly better anticoagulation treatment compliance than male patients 21 days postoperatively. However, there was no significant difference between patients of different ages, and patients younger than 65 appeared to take anticoagulant drugs in a timelier manner than older patients. In addition, patient compliance decreased with increasing follow-up time. Continuous follow-ups after discharge significantly improved patient compliance according to the treatment protocol.

**Table 3:** Compliance data of 180 patients undergoing THA/TKA

	Patients number/percent of good patient compliance. N (%)				
	Discharge time (days)				
	1	7	14	21	28
Patient age (years)					
< 65	81 (100)	81 (100)	79 (97.5)	77 (95.1)	74 (91.4)
> 65	99 (100)	94 (94.9)	90 (90.9)	85 (84.9)	80 (80.8)
* P* value	——	——	0.065	**0.041**	**0.045**
Sex					
male	63 (100)	62 (98.4)	58 (92.1)	55 (87.3)	50 (79.4)
female	117 (100)	116 (99.1)	114 (97.4)	111 (94.9)	108 (92.3)
* P* value	——	0.655	0.095	**0.019**	**0.011**
Receive a follow-up questionnaire					
Yes	180 (100)	179 (99.4)	175 (97.2)	172 (95.6)	168 (93.3)
No	180 (100)	172 (95.6)	163 (90.6)	151 (83.9)	143 (79.4)
* P* value	——	**0.018**	**0.008**	**0.000**	**0.000**

Good patient compliance was regarded as the agreement of taking their anticoagulant treatment protocol on time. It involves consuming patient drugs, the patient’s mental and physical state, patient and family member’s words. The bolded *p* value indicates statistical significance with *p* < 0.05.

### Patient satisfaction

**Fig. 1:**
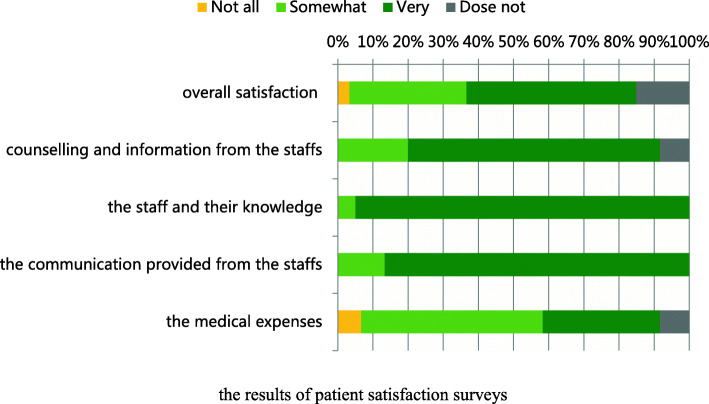
the results of patient satisfaction surveys

As shown in Fig. 1, the overall satisfaction rates with anticoagulant therapy, for counselling and information from medical workers and for staff communication and service were 81.67 %, 91.67 %, and 100 %, respectively. Twelve patients (6.67 %) expressed dissatisfaction with medical expenses.

## Discussion

VTE after major orthopaedic surgery in the lower extremities is a major cause of mortality and health care costs [6]. Numerous preventative measures and guidelines have been implemented to reduce VTE disease during the past 5–10 years [14–17]. Outpatient protocols and length of stay have been reduced, and rapid recovery after surgery has been developed during the same period [18]. However, there is a lack of effective solutions for addressing anticoagulation treatment for patients discharged after THA/TKA. Exploring the best medical resources and methods for outpatients on anticoagulant therapy is essential for improving medical quality. To the best of our knowledge, this study is the first to provide an easy, quick VTE assessment tool for extramural hospital THA/TKA patients to ensure that each patient possesses a tailored VTE prophylactic agent. Through these data, doctors could better guide the follow-up to different types of patients, focus on high-risk patients, and reduce the waste of medical resources. In fact, we could obtain relevant data and information through questionnaires and follow-up, which is very easy to implement and has almost no other cost except labour costs.

Previous studies indicated that the 30-day cumulative VTE incidence for TKA was 1.1–1.4 %, while the cumulative incidence for VTE in THA was 0.6–0.9 % [6, 7, 19]. Flevas et al. [20] reported that the symptomatic VTE rate during the first 3 months post orthopaedic surgery was in the range of 1.3–10 %. The median time of VTE occurrence was eleven days postoperatively for THA and three days for TKA [12]. Therefore, continuous follow-ups for 30 days after discharge could improve patient compliance, understanding of VTE disease, and satisfaction, as well as further reduce the incidence of VTE and medical costs. In previous research [21–23], the ratio of TKA to THA ranged from 1.3 to 1.5, the mean age ranged from 69.9 to 71.2 years, and the proportion of female sex ranged from 82.0 to 83.9 %. Le et al. [24] completed a protocol for meta-analysis with 97,677 patients and showed that aspirin was as effective as rivaroxaban in preventing VTE after THA or TKA. However, in terms of DVT, aspirin showed an increased risk compared with rivaroxaban. Consistent with most of the published studies [25–27], the results of anticoagulant measures after discharge indicated that new oral anticoagulants are mainly used outside the hospital. This may be due to the outstanding advantages of new anticoagulants, such as stable pharmacokinetics, fixed doses, and good safety.

Due to the traditional Chinese concept and the presence of more female patients, we found that female patients significantly exhibited better compliance with the anticoagulant treatment of VTE disease than male patients [28]. In addition, subjects over 65 years old had a worse understanding of the above information, which may affect the compliance of this kind of population. Of note, patients over 65 years old often had a much higher risk of having underlying diseases such as hypertension, diabetes, and coronary heart disease than younger patients. Thus, it is necessary to pay attention to the anticoagulation management of elderly patients. The study was a small-sample, single-centre survey, and the results need further research to confirm. The outcome showed that patient compliance might decrease with increasing follow-up time, while continuous follow-ups and guidance after discharge could improve this situation, especially after 14 days of discharge.

Implementing a multi-mode approach to venous thromboembolism prevention and a rapid recovery plan is beneficial to reducing the incidence of VTE [6, 29]. Patient educational programmes performed by clinical pharmacists have added a synergistic effect to decrease the risk of VTE [11]. Our findings may show that the current recommendations for VTE prophylaxis are adequate for preventing VTE in hospitals but inadequate after discharge. The optimal prophylaxis algorithm should be determined to allow for a safe and productive prophylaxis regimen in the setting of medical and surgical risk factors that might further decrease VTE incidence. Profoundly investigating the current status of anticoagulant therapies will contribute to further understanding VTE and the degree of satisfaction with medical services. This is a crucial approach to anticoagulant therapy.

The Chinese Guideline for Prevention of Venous Thromboembolism after Major Orthopaedic Surgery (2016 version) recommended that the duration of anticoagulant treatment be more than 10–14 days for patients undergoing THA/TKA, which was extended to 35 days for patients undergoing THA/TKA [2]. The survey results showed that most patients (93.3 %) were treated for 2–4 weeks, and only three patients were treated for less than 2 weeks. The follow-up appointment time for 85 % of patients was 14 days, which was convenient for observing the curative effect and patient condition in a timely manner and adjusting the treatment protocols.

In China, anticoagulant clinics, on-site services from community doctors, and new medical provider types such as telemedicine and network hospitals appeared successively. With national policies such as “medical reform,” “hierarchical medical system,” and “family doctors”, patients can enjoy the convenience of this science and technology anytime and anywhere [2]. However, instead of going to an anticoagulant clinic, having at-home visits from a nurse or pharmacist, or communicating remotely over the telephone, almost all patients preferred to continue to be treated in an orthopaedic clinic after discharge. This suggests that orthopaedic clinics are still the primary choice, which may be related to the patients’ lack of understanding and trust in new medical resources and approaches. This finding may also be associated with the nonuniform distribution of medical resources and the current immature healthcare-deliverystrategies in China. Therefore, the government or hospitals should reasonably arrange the schedule and distribution of medical resources to explore and optimize the new direction of management, which involves personalized customization, long-term follow-up, and therapy process monitoring. Furthermore, guiding and educating patients to make full use of the current medical technology and resources is also indispensable.

Adherence to treatment always depends on the individuals themselves for outpatient compliance. However, a considerable number of patients might discontinue anticoagulant therapy when they are discharged or after a short period of anticoagulant treatment. For such patients, health education, strengthening communication between them and their doctors, paying attention to the control of risk factors, and regular monitoring are helpful to improve the compliance with anticoagulation treatment and reduce the risk of bleeding [30]. The results of understanding VTE disease demonstrated that most enrolled patients knew basic disease information and were able to respond to different situations during VTE treatment. However, patients lacked additional knowledge that may affect treatment, safety, and compliance with anticoagulant therapy. Such information included side effects, the risks of not taking a suitable dose drug as required, the effectiveness of other medicines or the interaction of certain foods with the anticoagulant drugs. This suggests that patients should receive comprehensive education and guidance for anticoagulation management. These guidance measures cover basic education on anticoagulation treatment, mastery of drug properties, the risk and management of bleeding, remedial measures if doses are missed, exercise guidance, and medical support during anticoagulation.

Last, the present study has several limitations. Noticeably, this investigation is a randomized controlled study without double-blind randomization. The data collectors acquired the information of patients so that they could administer the appropriate interventions. However, we recognized that this could be a source of experimenter bias. Additionally, considering its relatively small sample size, we should adopt a conservative attitude regarding the interpretations of VTE prophylaxis outcomes. Another 3–6 months follow-up time may also be necessary to better evaluate the clinical efficacy for further research. Furthermore, our tools failed to stratify by patient comorbidities, which can be a significant confounding factor causing VTE disease.

In conclusion, the present study suggests that the quality of anticoagulant treatment for patients discharged after THA/TKA needs to be further improved in China. To help patients effectively and safely prevent VTE, we suggest that the government better improve medical resources and approaches, strengthen patient management and patient education, and optimize the patient experience. By comprehensive education and guidance of patients in understanding VTE, we can decrease its rates outside the hospital. Furthermore, we found that continuous follow-ups for 30 days after discharge, especially for patients older than 65, significantly improves VTE patient satisfaction and reduces the incidence of VTE and medical costs.

## Supplementary Information

**Additional file 1.**

## Data Availability

We claim that all relevant raw data and materials in this study are available. We also agreed that all datasets on which the paper’s conclusions rely should be available to readers. All data generated during this study are included in this published article. The datasets used during the current study available from the corresponding author on reasonable request.
