# Supplementary material for: Current status survey of the extramural hospital management of venous thromboembolism after total hip and knee arthroplasty in China
Source: BMC Musculoskelet Disord. 2021 Sep 13;22:787. doi: 10.1186/s12891-021-04663-1 (PMC8438984; doi:10.1186/s12891-021-04663-1)
Supplement: Supplementary file 1 — Additional file 1. [file 12891_2021_4663_MOESM1_ESM.docx]

**Appendix A：TKA/THA Patients’ Experience with Anticoagulation In post-discharge period: A Tool for Quality Improvement**

| 2 | 3 |
| --- | --- |

Please DO NOT write patient name or any other unique identifiers on the survey. Any responses will be kept CONFIDENTIAL. Participation is voluntary. Responses will be combined, and the information will remain ANONYMOUS. Please completely shade in each response oval that you select like this ●. In the boxes, print neatly and write only 1 digit per box, for example:

Do not cross out questions on the form that does not apply. A third part may collate and report data from your responses. Completion of this form provides permission to do this. Thank you for your participation!

Gender: □ Male □ Female Age: □□year (if 89 years or older, enter 89)

At discharge, I had an appointment scheduled within:

□ 7 days □ 14 days □21 days □28 days □ I didn’t have an appointment scheduled

My care is being managed by a(n):

| □Anticoagulation Clinic | □ Doctor’s Office |  |
| --- | --- | --- |
| □At home with visiting nurse/pharmacist |  |  |
| □Remotely by telephone | □ At another type of site | |

After discharge, I received these anti-clotting drugs (check all that apply):

| □ Heparin Sodium Injection  □Vitamin K Antagonist [e.g. warfartin® (Coumadin)] |
| --- |
| □New Oral Anticoagulant [e.g. Apixaban (Eliquis®), rivaroxaban (Xarelto®)] |
| □Injectables [e.g. enoxaparin (Lovenox®)], fondaparinux (Arixtra®), Dalteparin (Fragmin®), Nadroparin (Fraxiparine®)] |
| □Aspirin [e.g. Byaspirin] |
| □ Others |

I have been on my anti-clotting drug for: □ less than 3 days; □3days–1 week; □2–4 weeks; □4–5 weeks; □more than 5 weeks;

I told __ of my doctors (eye doctor, dentist, skin doctor,etc.) that I am on an anti-clotting drug. □ All □Some □No

I received helpful counseling and information about these specific facts (shade in all response ovals that apply):

| □ My diagnosis | □ Benefits of therapy |  |
| --- | --- | --- |
| □ Who to call if there is a problem | □ Activities to avoid |  |
| □ When to call the clinic or doctor | □ Signs and symptoms of bleeding |  |
| □ Side effects | □ What to do if miss a dose |  |
| □ How much drug to take and when | □ Importance of taking my medicine |  |
| □ How long I will need to take and when | □ What to do if I am having a dental or medical procedure |  |
| □ My medication list including any changes | □ Risks if I take too little or too much |  |
| □ Other anti-clotting drugs available for use in treating a clot | | |
| □The affect food can have on the effectiveness | | |
| □Other drugs and over-the counter products that can interact with my anti-clotting drug | | |
| Have you been admitted to an ER or hospital with taking your anti-clotting drug? □ Yes □ No | | |

| How satisfied are you: | Not all | Somewhat | Very | Dose not |
| --- | --- | --- | --- | --- |
| With your anti-clotting treatment? | □ | □ | □ | □ |
| With the counselling and information you received about treatment with your anti-clotting drug? | □ | □ | □ | □ |
| With the staff and their knowledge about your drug and how to monitor your drug? | □ | □ | □ | □ |
| If your drug required testing, with the testing procedure at the place where testing occurs? | □ | □ | □ | □ |
| With dose adjustments and instructions for your new dose when they occurred? | □ | □ | □ | □ |
| With the communication provided from my doctor about my diagnosis, drug therapy, and tests that may be required? | □ | □ | □ | □ |
| With the cost of my anti-clotting drug? | □ | □ | □ | □ |
